# Age-related reflex responses from peripheral and central chemoreceptors in healthy men

**DOI:** 10.1007/s10286-014-0263-9

**Published:** 2014-11-25

**Authors:** Bartłomiej Paleczny, Piotr Niewiński, Agnieszka Rydlewska, Massimo F. Piepoli, Ludmiła Borodulin-Nadzieja, Ewa A. Jankowska, Beata Ponikowska

**Affiliations:** 1 Department of Physiology, Wroclaw Medical University, ul. Chałubińskiego 10, 50-368 Wroclaw, Poland; 2 Department of Cardiology, Centre for Heart Diseases, 4th Military Hospital, Wroclaw, Poland; 3 Department of Heart Diseases, Wroclaw Medical University, Wroclaw, Poland; 4 Heart Failure Unit, Cardiac Department, G. da Saliceto Hospital, AUSL Piacenza, Piacenza, Italy; 5 Laboratory for Applied Research on Cardiovascular System, Department of Heart Diseases, Wroclaw Medical University, Wroclaw, Poland

**Keywords:** Ageing, Chemoreceptors, Baroreceptors, Heart rate Variability, Autonomic status

## Abstract

**Objective:**

The study aimed: (i) to characterize reflex responses from peripheral and central chemoreceptors in different age groups of healthy men (<50 years old vs ≥50 years old) and, (ii) to assess, within these groups, whether there is any relationship between ventilatory and hemodynamic responses from chemoreceptors and indices of autonomic nervous system (ANS).

**Methods:**

Peripheral chemoreflex sensitivity was assessed by the transient hypoxia method and respiratory, heart rate (HR) and blood pressure responses were calculated. Central chemoreflex sensitivity was assessed by the rebreathing method and respiratory response was calculated. ANS was assessed using heart rate variability indices and baroreflex sensitivity (BRS).

**Results:**

Sixty-seven healthy men were divided into 2 groups: <50 years (*n* = 38, mean age: 32 ± 10 years) and ≥50 years (*n* = 29, mean age: 61 ± 8 years). There were no differences in respiratory response from central and peripheral chemoreceptors between the older and younger groups of healthy males. We found a significantly different pattern of hemodynamic responses from peripheral chemoreceptors between the older and the younger groups. The former expressed attenuated HR acceleration and exaggerated blood pressure increase in response to transient hypoxia. Blunted HR response was related to reduced BRS and sympathovagal imbalance characterized by reduced vagal tone. Blood pressure responses seemed to be independent of sympathovagal balance and BRS.

**Interpretation:**

Ageing impacts hemodynamic rather than respiratory response from chemoreceptors. Impaired arterial baroreflex and sympathovagal imbalance related to ageing may contribute to decreased heart rate response, but not to increased blood pressure response from peripheral chemoreceptors.

## Introduction

Cardiopulmonary reflex control via autonomic nervous system (ANS) primarily comprises neural pathways from: *arterial baroreceptors* (primary blood pressure sensors), *brainstem central chemoreceptors* (primary CO_2_/H^+^ sensors) and *peripheral arterial chemoreceptors* (primary O_2_ sensors) [1–3]. It has been widely documented that impaired autonomic cardiopulmonary regulation (as evidenced by decreased arterial baroreflex sensitivity and augmented central or peripheral chemosensitivity) characterizes numerous pathological conditions, including cardiovascular disorders [2, 4], diabetes [5], obstructive sleep apnea [6], and metabolic syndromes [7]. In particular, these abnormalities in the context of cardiac diseases may play an important, pathophysiological role, and provide diagnostic information [8-10]. Surprisingly, physiological alterations in these mechanisms in healthy aging individuals have not been sufficiently evaluated. While it has been shown that aging is associated with reduced arterial baroreflex sensitivity [11], previous studies on age-related changes in both central and peripheral chemosensitivity provided discordant results [12, 13] and mainly focused on the evaluation of respiratory response to chemoreceptors stimulation [14–16]. Data on hemodynamic responses to hypoxia/hypercapnia are scarce [12, 17, 18]. Likewise, to the best of the our knowledge, none of the previous works assessed the relationship between reflex responses from chemoreceptors and indices of ANS in the context of aging. Moreover, a growing body of evidence suggests that peripheral chemoreceptor hypersensitivity may be a key player in the pathogenesis of various sympathetically mediated disease. In addition, exaggerated hemodynamic components of the peripheral chemoreflex, seems to be an underlying mechanism, and not the respiratory components [19]. As ageing is associated with sympathetic predominance [20] it may well be expected that aging is also associated with augmented response from peripheral chemoreceptors.

Thus, we undertook the study: (i) to characterize reflex responses from peripheral and central chemoreceptors in different age groups of healthy men (<50 vs ≥50 years old) and, (ii) to assess, within these groups, whether there is any relationship between ventilatory and hemodynamic responses from chemoreceptors and autonomic indices [such as heart rate variability (HRV) and baroreflex sensitivity (BRS)].

### Study population

The study was performed at the Department of Cardiology, 4th Military Hospital (Wroclaw, Poland). Healthy male volunteers, aged between 20 and 80 years participated in the study. Exclusion criteria were: (1) smoking, (2) history of any acute or chronic illness, (3) history of alcohol or drug addiction, and (4) any drug therapy. The study protocol was approved by the local ethics committee. All subjects gave an informed consent. The study was conducted in accordance with the Helsinki Declaration.

### Study protocol

Prior to the autonomic testing all subjects underwent a physical examination. All subjects were asked to refrain from beverages with caffeine for at least 12 h before the study. Autonomic testing was performed between 9:00 and 11:00 a.m., in the quiet, light-attenuated room with stable ambient temperature ~22 °C.

Study protocol consisted of the five stages performed in the following order: (1) familiarization (at least 5 min), (2) resting (15 min), (3) evaluation of peripheral chemoreceptors (with transient hypoxia method—see below, ~40 min), (4) resting with controlled breathing (5 min), and (5) evaluation of central chemoreceptors (with rebreathing method—see below, ~20 min). Stages 1–4 were carried out with subjects lying supine on a bed, attached to the equipment described below (section: “Study equipment”, point “A”). Before the final stage subjects were asked to sit on a chair, the study equipment was replaced (section: “Study equipment”, point “B”) and examination was carried out with subjects sitting in an upright position.

### Study equipment

#### A. *Stages 1*–*4*

Hemodynamic parameters: systolic blood pressure (SBP, mmHg), diastolic blood pressure (DBP, mmHg), mean arterial pressure (MAP, mmHg), stroke volume (SV, mL/beat), cardiac output (CO, L/min) and systemic vascular resistance (SVR, dyn x s/cm^5^) were measured continuously and non-invasively using a cardiovascular monitor (Nexfin, BMEYE B.V., Amsterdam, Netherlands) with continuous, non-invasive, digital recording of finger arterial pressure. ECG wave was recorded using ECG module for Nexfin (Nexfin, BMEYE B.V., Amsterdam, Netherlands). Heart rate (HR, bpm) was calculated from ECG recording.

Subjects breathed through the oro-nasal face mask (Hans Rudolph, Inc., Shawnee, KS, USA) connected with a two-way non-rebreathing T-shape valve (Hans Rudolph, Inc.). Respiratory parameters: breathing rate (BR, breaths/min) and tidal volume (V_T_, L/breath) were measured continuously using differential pressure transducer (FE141 Spirometer, ADInstruments, Sydney, Australia) with 1,000 L/min flowhead (MLT3000L, ADInstruments). Minute ventilation (MV, L/min) was calculated from BR and V_T_. End-tidal carbon dioxide concentration (etCO_2_, mmHg) was measured continuously using capnograph (Capstar 100, CWE Inc., Ardmore, PA, USA). Blood oxygen saturation (SpO_2_, %) was measured continuously using pulse oximeter (Masimo Corporation Irvine, CA, USA) with probe placed on the subject’s earlobe.

During stage 3 (evaluation of peripheral chemoreceptors with transient hypoxia method—see below) pure nitrogen (class 5.2), stored in a 10 L gas cylinder, was administrated via the small-diameter tube placed inside a large-diameter breathing tube (ADInstruments) connected directly with a face mask. Nitrogen was administrated with a flow rate > 15 L/s regulated to the nearest 0.1 L using flowmeter. As shown by the previous papers from our lab [21–23], short nitrogen administration with a high flow rate to the breathing tube causes a transient nitrogen accumulation inside the tube sufficient to evoke a substantial desaturation.

Data acquisition device (PowerLab 16/30, ADInstruments) and laptop (Dell Inc., Round Rock, TX, USA) with data acquisition and analysis software (LabChart 7, ADInstruments) were used for data acquisition. All data were recorded and stored at sampling frequency of 1 kHz (16-bit resolution).

#### B. *Stage 5*

Subjects breathed through the oro-nasal face mask (Hans Rudolph, Inc.) with mouthpiece, connected with three-way T-shape inflatable balloon-type valve (Hans Rudolph, Inc.) with two arms. Lower arm was connected with a reservoir bag pre-filled with 5 L of 100 % oxygen. Upper arm opened outside. Simultaneous closing of upper arm and opening of lower arm created a closed circuit limited to mouthpiece, valve and 5-L bag. BR, V_T_ and etCO_2_ were measured breath-by-breath using gas exchange analyzer (Ultima CPX, Medical Graphics Corporation, St. Paul, Minnesota, USA). MV was calculated from BR and V_T_.

Data were stored on a PC computer with data acquisition and analysis software (Medical Graphics Corporation, Saint Paul, MN, USA).

### Assessment of resting hemodynamic and respiratory parameters

Resting values of hemodynamic and respiratory parameters were defined as arithmetic averages from 10-min period of an acceptable quality selected from 15-min resting stage recording. The same 10-min period was used for calculation of HRV indices and BRS using the sequence method (BRS-Seq, ms/mmHg) [24] and the spectral method (BRS-αLF and BRS-αHF, ms/mmHg) [25].

### Assessment of heart rate variability

HRV describes fluctuation in RR intervals duration and provides a quantitative evaluation of the sympathovagal interaction modulating cardiovascular function [26, 27]. The following HRV indices were used:

(i) Time-domain analysis: RMSSD (ms), the square root of the mean of the sum of the squares of differences between adjacent NN intervals; pNN50 (%), number of pairs of adjacent NN intervals differing by more than 50 ms divided by the total number of all NN intervals; RMSSD and pNN50 reflect short-term variability of RR intervals related to parasympathetic tone [27];
(ii) Frequency-domain analysis: LF (ms^2^), low frequency range (0.04–0.15 Hz) of HRV spectrum; HF (ms^2^), high frequency range (0.15–0.4 Hz) of HRV spectrum; LF/HF, low frequency HRV to high frequency HRV ratio [27].

Standard autoregressive methods [26, 28] were used to calculate frequency-domain HRV parameters. High frequency band of HRV is interpreted as a marker of efferent vagal activity. Physiological basis of the low frequency component of HRV is more controversial. LF includes mainly sympathetic or mixed sympathetic and parasympathetic influences [27].

### Assessment of cardiac baroreflex sensitivity

Cardiac baroreflex sensitivity (BRS) was evaluated by:

(i) The sequence method: BRS is calculated based on selected sequences of three consecutive heart beats where: (i) increase in RR interval duration by at least 5.0 ms is accompanied by simultaneous increase in SBP by at least 1.0 mmHg or, (ii) decrease in RR duration interval by at least 5.0 ms is accompanied by simultaneous decrease in SBP by at least 1.0 mmHg; BRS is expressed as an average slope of all regression lines linking RR intervals duration to SBP for all sequences selected (BRS-Seq, ms/mmHg) [24];
(ii) The spectral (or α-indices) method: BRS is estimated based on spectral analysis of HRV and blood pressure variability (BPV) and expressed as a ratio of HRV to BPV within two frequency bands: low frequency band (0.04–0.15 Hz) (BRS-αLF, ms/mmHg) and high frequency band (0.15–0.40 Hz) (BRS-αHF, ms/mmHg) [24, 25];
(iii) The controlled breathing method: based on the synchronization of the imposed breathing rate (6 breaths/min) with rhythmical oscillations in HR and SBP; BRS is calculated by dividing an average amplitude of RR interval duration by an average amplitude of SBP (BRS-CB, ms/mmHg) [28].

The spectral method allows for separate assessment of the sympathetic and parasympathetic contribution to baroreflex regulation of heart rate (reflected by BRS-αLF and BRS-αHF, respectively). The sequence method, however, is considered as a tool for “comprehensive” evaluation of baroreflex modulation of the sinus node, as this modulation is suggested to be wider than that explored by the BRS-αLF and BRS-αHF parameters [29]. The controlled breathing method is suggested as the method of choice for BRS assessment by some papers, as this is the only one which removes potentially confounding influence of breathing rate and is characterized by the highest reproducibility. Thus, BRS-CB was also included [28].

BRS-CB was estimated based on HR and SBP recording obtained during 5-min of the controlled breathing stage (stage 4), while subjects were asked to adjust the breathing rhythm to the rhythm of the metronome presented on the laptop monitor (6 breaths/min). 200-s period of an acceptable quality was selected from 5 min of HR and SBP recording to calculate BRS-CB.

### Assessment of central chemoreflex sensitivity

Central chemoreflex sensitivity was assessed using the rebreathing method [30]. The test consisted of three consecutive phases: (i) resting phase (at least 3 min), (ii) rebreathing phase (usually 5–10 min), and, (iii) recovery phase (at least 3 min). During the resting and recovery phases, subjects breathe in room air. At the end of the resting phase, subject was switched from breathing in room air to breathing within a closed circuit limited to valve, mouthpiece, and reservoir bag pre-filled with 5 L of 100 % oxygen. Rebreathing within a closed circuit caused an accumulation of carbon dioxide in expired and inspired gas mixture resulting in a steady increase in carbon dioxide concentration in the blood stream. Progressive hypercapnia led to reflexive increase in respiratory activity (reflected in MV increase). Rebreathing phase lasted until the subject signalled breathlessness or etCO_2_ exceeded 70 mmHg. Slope of the regression line relating increase in MV to increase in etCO_2_ during the rebreathing phase was interpreted as a measure of central chemoreflex sensitivity in terms of respiratory response (CChS-Ve, L/min/mmHg).

### Assessment of peripheral chemoreflex sensitivity

Peripheral chemoreflex sensitivity was assessed using transient hypoxia method [21, 31, 32]. Two components of hypoxia-induced chemoreflex response were measured: (i) respiratory response (reflected in MV increase) and (ii) hemodynamic responses: heart rate acceleration (reflected in HR increase) and blood pressure response (reflected in SBP increase). During the test, several (up to 7) short (lasting 5–40 s) administrations of pure nitrogen into inspired air were performed to achieve falls in SpO_2_ with maximal desaturation varying from 65 to 85 % (conditions of short hypoxemic hypoxia). The length of consecutive nitrogen administrations was adjusted ad hoc based on the fall in SpO_2_ caused by the first 10-s nitrogen administration. Nitrogen administrations were separated at least by 5-min periods of room air breathing. Calculations as follows were performed for each nitrogen administration.

*Respiratory response* 35-s period of recording starting from the end of nitrogen administration were selected. Within this time frame the highest three consecutive MV values and the lowest SpO_2_ value were isolated. Then, isolated MV values were averaged and plotted against the isolated SpO_2_ value providing Point 1. Baseline values of MV and SpO_2_ were defined as arithmetic averages from 90-s period preceding nitrogen administration. Then, baseline MV average was plotted against SpO_2_ average providing Point 2. Slope of the regression line linking Point 1 and Point 2 were obtained. As the value of the slope was negative, we used its absolute value for subsequent calculations. Arithmetic averages of absolute values of the slopes for all nitrogen administrations was interpreted as a measure of peripheral chemoreflex sensitivity in terms of respiratory response (PChS-Ve, L/min/%).

*Hemodynamic responses* Calculations of peripheral chemoreflex sensitivity in terms of heart rate response (PChS-HR, bpm/%) and systolic blood pressure response (PChS-SBP, mmHg/%) were performed analogously as in the assessment of respiratory response described above with two differences: (i) 55-s period of recording starting from the end of nitrogen administration were selected (instead of 35-s period), (ii) the highest value of HR (for PChS-HR) and the highest value of SBP (for PChS-SBP) were isolated (instead of the highest three consecutive MV values).

Detailed description of data processing was presented before [21].

### Data and statistical analyses

Data and statistical analyses were conducted using Statistica 10 (Statsoft, Tulsa, OK, USA) and MATLAB (MathWorks, Natick, MA, USA). Variables were presented as a mean ± standard deviation (for normally distributed variables) or median with lower and upper quartile (for skewed distributed variables). To normalize the distribution, skewed distributed variables were log transformed (natural logarithm, (ln)). Unpaired Student *t* test was used to test the inter-group differences.

Pearson’s linear correlation coefficient was used to assess the relations between variables. *P* value <0.05 was considered statistically significant.

## Results

The sixty-seven healthy men who participated in the study were divided into 2 groups: <50 years (*n* = 38, mean age: 32 ± 10 years) and ≥50 years (*n* = 29, mean age: 61 ± 8 years).

### Resting hemodynamic and respiratory parameters

The older group (≥50 years) was characterized by reduced SV, CO and elevated SVR as compared to those <50 years. There were no differences between groups in the remaining hemodynamic and all respiratory parameters at rest (Table 1).

**Table 1:** Characteristics of examined healthy men

	Men <50 years old (*n* = 38)	Men ≥50 years old (*n* = 29)
Anthropometric parameters		
Height (cm)	181 ± 6	175 ± 6***
Weight (kg)	83 ± 13	85 ± 12
BMI (kg/m^2^)	25.3 ± 3.9	27.8 ± 3.9**
Resting hemodynamic parameters		
HR (bpm)	65 ± 10	68 ± 11
SBP (mmHg)	119 ± 15	122 ± 13
DBP (mmHg)	70 ± 9	70 ± 8
MAP (mmHg)	88 ± 11	91 ± 9
SV (mL/beat)	108 ± 13	87 ± 15***
CO (L/min)	7.0 ± 0.9	5.9 ± 1.3***
SVR (dyn x s/cm^5^)	1,031 ± 190	1,303 ± 379***
Resting respiratory parameters		
BR (breaths/min)	14 ± 5	13 ± 4
MV (L/min)	11.3 ± 3.3	11.5 ± 4.1
etCO_2_ (mmHg)	37 ± 6	35 ± 5
SpO_2_ (%)	97 ± 1	96 ± 2
Heart rate variability		
RMSSD (ms)	44 ± 19	28 ± 15***
pNN50 (%)	13 ± 9	5 ± 6***
LF (ms^2^)	210 (138; 432)	311 (228; 812) *
HF (ms^2^)	343 (297; 457)	271 (189; 370)***
LF/HF	0.60 (0,60; 1,23)	1.17 (0,60; 3,29)***
Cardiac baroreflex sensitivity		
BRS-Seq (ms/mmHg)	14.6 ± 6.2	8.3 ± 3.9***
BRS-αLF (ms/mmHg)	0.70 (0.56; 0.94)	0.78 (0.68; 0.89)
BRS-αHF (ms/mmHg)	1.32 (1.19; 1.53)	1.10 (0.99; 1.24)***
BRS-CB (ms/mmHg)	10.3 ± 5.5	6.5 ± 3.2**
Chemoreflex sensitivity		
CChS-Ve (L/min/mmHg)	0.63 ± 0.50	0.78 ± 0.57
PChS-Ve (L/min/%)	0.39 (0.27; 0.55)	0.30 (0.19; 0.52)
PChS-HR (bpm/%)	0.61 ± 0.30	0.42 ± 0.17**
PChS-SBP (mmHg/%)	0.55 ± 0.33	0.72 ± 0.22*

Data are presented as a mean ± standard deviation or median with lower and upper quartile where appropriate

*HR* heart rate, *SBP* systolic blood pressure, *DBP* diastolic blood pressure, *MAP* mean arterial pressure, *SV* stroke volume, *CO* cardiac output, *SVR* systemic vascular resistance, *BR* breathing rate, *MV* minute ventilation, *etCO*_*2*_ end-tidal CO_2_ concentration, *SpO*_*2*_ blood oxygen saturation, *RMSSD* the square root of the mean of the sum of the squares of differences between adjacent NN intervals, *pNN50* number of pairs of adjacent NN intervals differing by more than 50 ms divided by the total number of all NN intervals, *LF* low frequency range of HRV spectrum, *HF* high frequency range of HRV spectrum, *LF/HF* low frequency HRV to high frequency HRV ratio, *BRS-Seq* cardiac baroreflex sensitivity assessed by the sequence method, *BRS-αLF* cardiac baroreflex sensitivity in a low frequency range assessed by the spectral method, *BRS-αHF* cardiac baroreflex sensitivity in a high frequency range assessed by the spectral method, *BRS-CB* cardiac baroreflex sensitivity assessed by the controlled breathing method, *CChS-Ve* central chemoreflex sensitivity in terms of respiratory response, *PChS-Ve* peripheral chemoreflex sensitivity in terms of respiratory response, *PChS-HR* peripheral chemoreflex sensitivity in terms of heart rate response, *PChS-SBP* peripheral chemoreflex sensitivity in terms of systolic blood pressure response

* *p* < 0.05; ** *p* < 0.01; *** *p* < 0.001

#### Heart rate variability

**Fig. 1:**
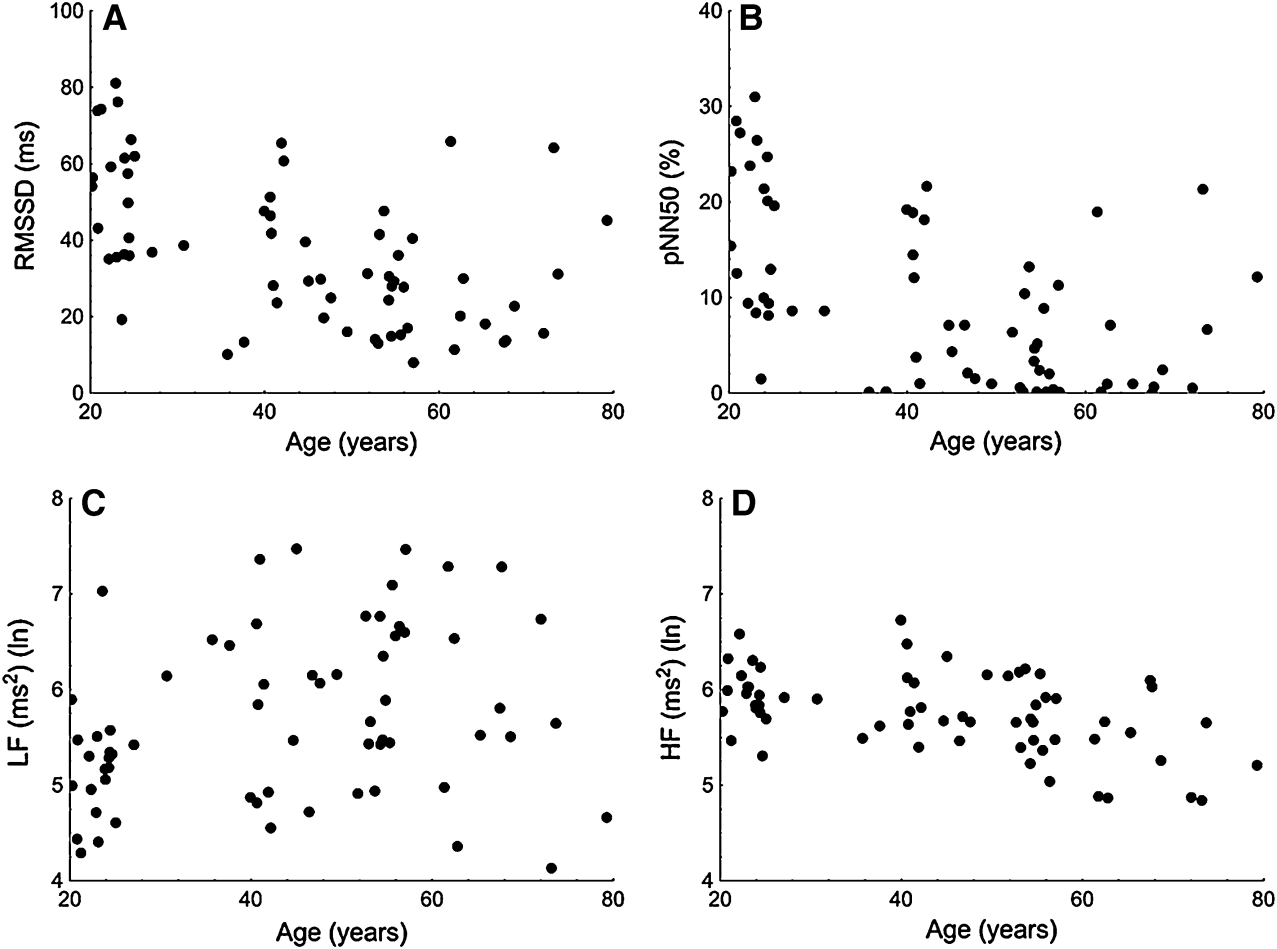
Graph relating age to the following heart rate variability measures: **a** the square root of the mean of the sum of the squares of differences between adjacent NN intervals (RMSSD), **b** the number of pairs of adjacent NN intervals differing by more than 50 ms divided by the total number of all NN intervals (pNN50), **c** the log of low frequency range of HRV spectrum (LF) and **d** the log of high frequency range of HRV spectrum (HF); (ln), natural logarithm

In the older group, significant reduction in the parasympathetic-related HRV indices (RMSSD, pNN50, HF) and an increase in sympathetic-related indices (LF, LF/HF) were observed. (Table 1; Fig. 1).

#### Cardiac baroreflex sensitivity

**Fig. 2:**
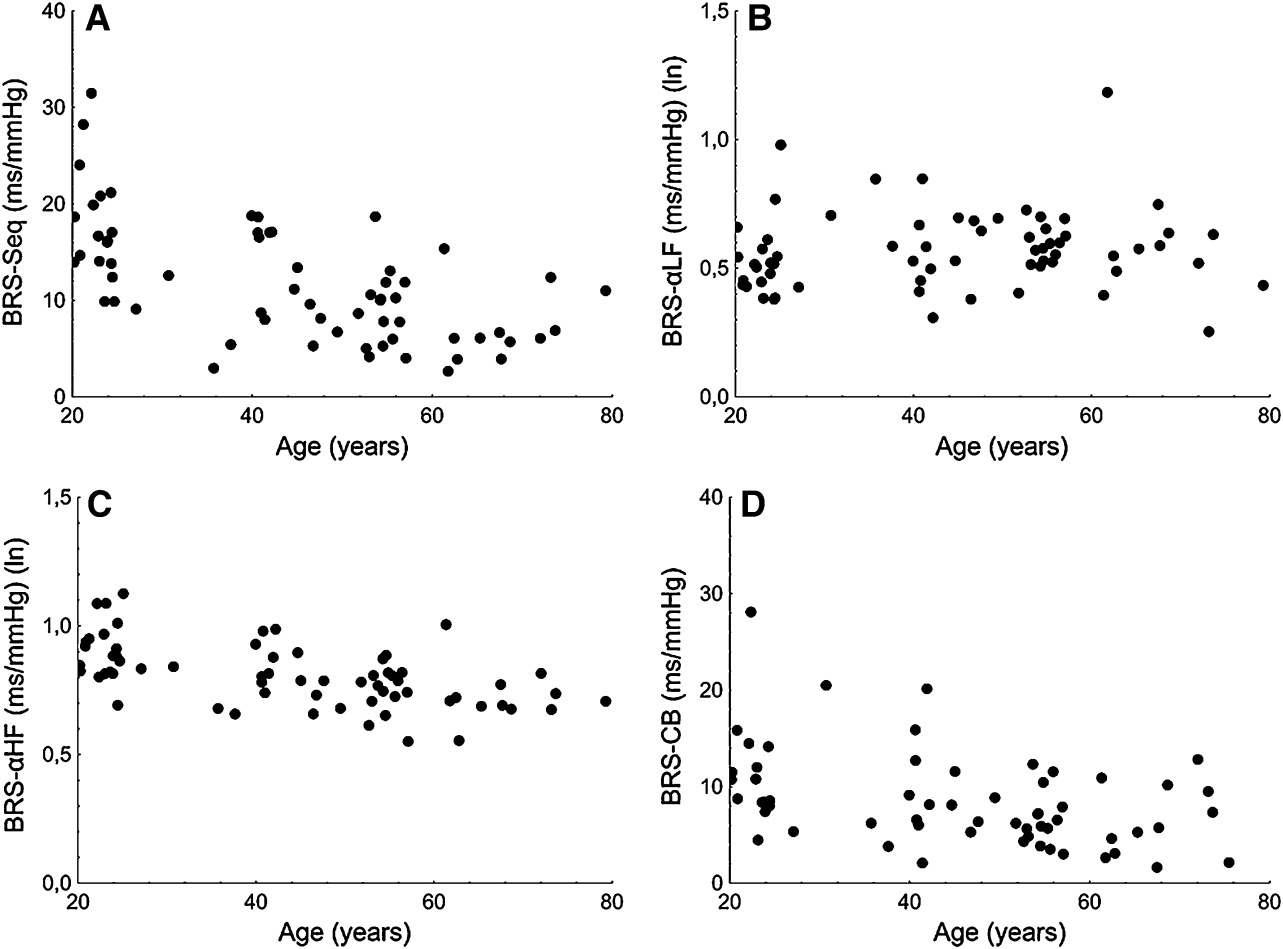
Graph relating age to the following baroreflex sensitivity measures: **a** cardiac baroreflex sensitivity assessed by the sequence method (BRS-Seq), **b** cardiac baroreflex sensitivity in a low frequency range assessed by the spectral method (BRS-αLF), **c** cardiac baroreflex sensitivity in a high frequency range assessed by the spectral method (BRS-αHF) and **d** cardiac baroreflex sensitivity assessed by the controlled breathing method (BRS-CB); (ln), natural logarithm

All parameters of BRS, except for BRS-αLE, were significantly lower in men ≥50 years (Table 1; Fig. 2).

#### Chemoreflex sensitivity

**Fig. 3:**
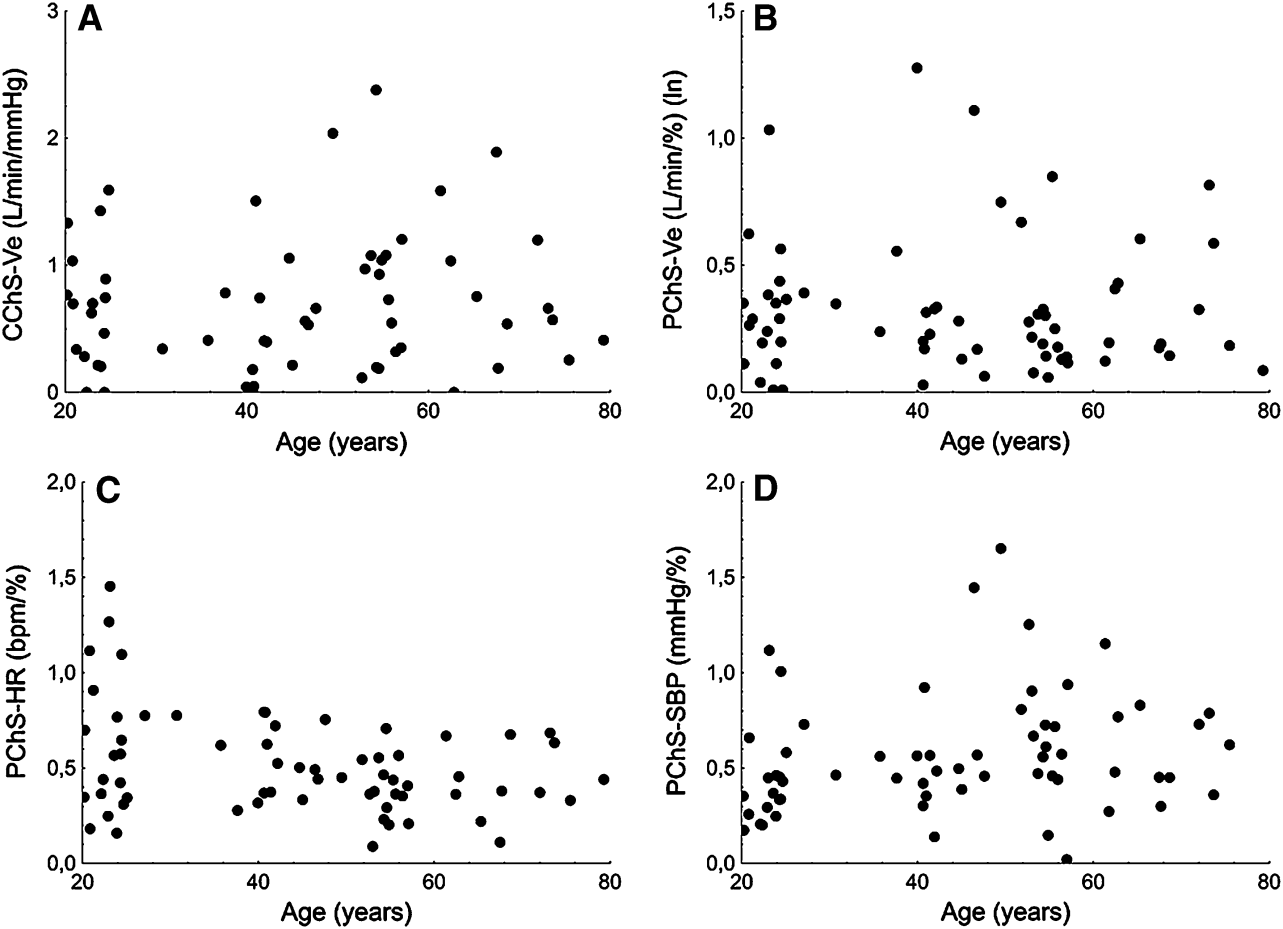
Graph relating age to the following chemoreflex sensitivity measures: **a** central chemoreflex sensitivity in terms of respiratory response (CChS-Ve), **b** peripheral chemoreflex sensitivity in terms of respiratory response (PChS-Ve), **c** peripheral chemoreflex sensitivity in terms of heart rate response (PChS-HR), **d** peripheral chemoreflex sensitivity in terms of systolic blood pressure response (PChS-SBP); (ln), natural logarithm

There were no differences in respiratory response from central (CChS-Ve) and peripheral chemoreceptors (PChS-Ve) between two groups. However, significant differences were found in hemodynamic responses from peripheral chemoreceptors. The older group reacted to transient hypoxia with a lower heart rate acceleration, but greater systolic blood pressure increase than the younger group (Table 1; Fig. 3).

#### Relations between responses from chemoreceptors and HRV and BRS

In the younger group, systolic blood pressure response to hypoxia correlated negatively with BRS-Seq (*r* = −0.42; *p* < 0.05) and BRS-αHF (*r* = −0.44; *p* < 0.05). There were no other significant correlations within this group (Table 2).

**Table 2:** Correlations between chemosensitivity and HRV and BRS in men <50 years old

	CChS-Ve	PChS-Ve	PChS-HR	PChS-SBP
	(L/min/mmHg)	(L/min/%)	(bpm/%)	(mmHg/%)
RMSSD (ms)	−0.23	0.08	0.19	−0.34
pNN50 (%)	−0.27	0.10	0.17	−0.28
LF (ln)	0.12	−0.26	−0.29	−0.14
HF (ln)	−0.18	0.29	−0.13	−0.12
LF/HF (ln)	0.19	−0.33	−0.23	−0.08
BRS-Seq (ms/mmHg)	−0.29	0.10	0.21	−0.42*
BRS-αLF (ln)	0.28	−0.23	−0.20	−0.08
BRS-αHF (ln)	−0.32	0.07	0.06	−0.44*
BRS-CB (ms/mmHg)	−0.31	−0.08	−0.03	−0.24

*r* Pearson’s linear correlation coefficients are presented, *RMSSD* the square root of the mean of the sum of the squares of differences between adjacent NN intervals, *pNN50* number of pairs of adjacent NN intervals differing by more than 50 ms divided by the total number of all NN intervals, *LF* low frequency range of HRV spectrum, *HF* high frequency range of HRV spectrum, *LF/HF* low frequency HRV to high frequency HRV ratio, *BRS-Seq* cardiac baroreflex sensitivity assessed by the sequence method, *BRS-αLF* cardiac baroreflex sensitivity in a low frequency range assessed by the spectral method, *BRS-αHF* cardiac baroreflex sensitivity in a high frequency range assessed by the spectral method, *BRS-CB* cardiac baroreflex sensitivity assessed by the controlled breathing method, *CChS-Ve* central chemoreflex sensitivity in terms of respiratory response, *PChS-Ve* peripheral chemoreflex sensitivity in terms of respiratory response, *PChS-HR* peripheral chemoreflex sensitivity in terms of heart rate response, *PChS-SBP* peripheral chemoreflex sensitivity in terms of systolic blood pressure response, (*ln*) natural logarithm

* *p* < 0.05; ** *p* < 0.01; *** *p* < 0.001

In the older group, respiratory response to hypercapnia correlated positively with BRS-αHF (*r* = 0.54; *p* < 0.05) and respiratory response to hypoxia correlated negatively with LF (*r* = −0.53; *p* < 0.01) and LF/HF (*r* = −0.48; *p* < 0.05). Heart rate response to hypoxia correlated with time-domain HRV indices (RMSSD: *r* = 0.53; *p* < 0.01 pNN50 %: *r* = 0.52; *p* < 0.01) and BRS parameters (BRS-CB: *r* = 0.42; *p* < 0.05; BRS-αLF: *r* = −0.52; *p* < 0.01). There were no other significant correlations within this group (Table 3).

**Table 3:** Correlations between chemosensitivity and HRV and BRS in men ≥50 years old

	CChS-Ve	PChS-Ve	PChS-HR	PChS-SBP
	(L/min/mmHg)	(L/min/ %)	(bpm/ %)	(mmHg/ %)
RMSSD (ms)	0.06	0.32	0.53**	−0.01
pNN50 (%)	0.04	0.35	0.52**	0.07
LF (ln)	0.12	−0.53**	−0.36	−0.05
HF (ln)	0.17	0.02	−0.28	−0.08
LF/HF (ln)	0.05	−0.48*	−0.22	0.01
BRS-Seq (ms/mmHg)	0.27	0.21	0.33	−0.29
BRS-αLF (ln)	−0.09	−0.36	−0.52**	−0.08
BRS-αHF (ln)	0.54**	−0.14	0.12	−0.25
BRS-CB (ms/mmHg)	0.15	0.05	0.42*	−0.19

*r* Pearson’s linear correlation coefficients are presented, *RMSSD* the square root of the mean of the sum of the squares of differences between adjacent NN intervals, *pNN50* number of pairs of adjacent NN intervals differing by more than 50 ms divided by the total number of all NN intervals, *LF* low frequency range of HRV spectrum, *HF* high frequency range of HRV spectrum, *LF/HF* low frequency HRV to high frequency HRV ratio, *BRS-Seq* cardiac baroreflex sensitivity assessed by the sequence method, *BRS-αLF* cardiac baroreflex sensitivity in a low frequency range assessed by the spectral method, *BRS-αHF* cardiac baroreflex sensitivity in a high frequency range assessed by the spectral method, *BRS-CB* cardiac baroreflex sensitivity assessed by the controlled breathing method, *CChS-Ve* central chemoreflex sensitivity in terms of respiratory response, *PChS-Ve* peripheral chemoreflex sensitivity in terms of respiratory response, *PChS-HR* peripheral chemoreflex sensitivity in terms of heart rate response, *PChS-SBP* peripheral chemoreflex sensitivity in terms of systolic blood pressure response, (*ln*) natural logarithm

* *p* < 0.05; ** *p* < 0.01; *** *p* < 0.001

There were no significant correlations between PChS-HR and PChS-SBP in either group.

## Discussion

The major new findings are: (i) no difference in respiratory response from central and peripheral chemoreceptors between the older and younger groups of healthy males, and, (ii) significantly different patterns of hemodynamic responses from peripheral chemoreceptors between the older and the younger groups. The former expressed attenuated heart rate acceleration and exaggerated blood pressure increase in response to transient hypoxia. Blunted heart rate response was related to reduced cardiac baroreflex sensitivity and sympathovagal imbalance characterized by reduced vagal tone. Interestingly, blood pressure response seemed to be independent of sympathovagal balance and baroreflex sensitivity within this group, (iii) additionally we confirmed previous findings that ageing is related to changes in autonomic balance in favour of sympathetic predominance and/or vagal withdrawal.

Previous studies on age-dependent changes in central chemosensitivity provided discordant results. The lack of differences between the younger and the older men in respiratory response from central chemoreceptors found in this study stays in agreement with findings of Chapman and Cherniack [13], Rubin et al. [15] and van Klaveren and Demedts [16], and contrasts with attenuation of this response in the elderly reported by others [12, 14, 35]. Garcia-Rio et al. [36] found no differences between the age groups in magnitude of respiratory response, while CO_2_ sensitivity threshold (end-tidal CO_2_ value sufficient to activate central chemoreflex) was reduced in the subjects aged 65–69 years and 70–74 years as compared with the younger. Thus, one possible explanation of such heterogenic results is that ageing impacts not the sensitivity of this reflex mechanism (magnitude of response to “abnormal” end-tidal CO_2_), but rather the set point around which this mechanism operates (range of end-tidal CO_2_ which is perceived as “abnormal”). Additional factors may include: relatively small samples studied and combining data from male and female. Taking into account that our paper investigates a sizeable sample of healthy males, we believe that there are no age-related differences in central chemosensitivity (as evaluated with rebreathing method) in this population.

Preserved respiratory response from peripheral chemoreceptors in the middle-aged and elderly men as observed in this study, was also described by other authors [16, 37, 38]. Contrary to that, others reported a decrease [12, 35, 36, 39, 40] or even increase [13, 17] with age in the respiratory component of peripheral chemoreflex. Two recent studies of Garcia-Rio et al. [36] and Lhuissier et al. [17] conducted in the relatively large samples of healthy subjects (*n* = 112 and *n* = 4 675, respectively) provided, probably, the most relevant attempts to clarify this issue. Garcia-Rio et al. [36] observed gradual decrease in respiratory response to progressive hypoxia from the age group 20–40 years to age groups 65–69 years and 75–79 years. There was no significant difference between the subjects aged 75–79 and 80–84 years, suggesting that this response stabilizes after the age of 75. In the work of Lhuissier et al. [17] respiratory response to hypoxia was elevated in the men aged >60 years as compared with the men aged <30 years. One possible explanation of this discrepancy is that Garcia-Rio et al. [36] used the mouth occlusion pressure (P_0.1_) instead of minute ventilation to assess ventilatory response from central and peripheral chemoreceptors. P_0.1_ reflects central inspiratory drive and is believed to be less influenced by age-related changes in lung structure and respiratory mechanics than minute ventilation.

Data analyzing the possible effects of aging on the hemodynamic component of peripheral chemoreflex are scarce. Blunted hypoxia-induced heart rate acceleration in the elderly was reported by Kronenberg and Drage [12] and Lucy et al. [18]. In the study of Lhuissier et al. [17] heart rate increase in response to hypoxia was reduced in the older men (age groups: 40–49, 50–59 and >60 years) as compared with the men aged <30 years. Thus, our results stay in line with these studies. To the best of our knowledge there were no published studies on age-related changes in systolic blood pressure response to transient hypoxia. In this study, relations between chemosensitivity and “markers” of (i) sympathovagal balance within cardiovascular system and, (ii) cardiac baroreflex sensitivity were assessed to find out if such changes in function of the autonomic nervous system may be associated with age-dependent changes in chemoreceptors activity.

In the group of men ≥50 years old, robust correlations between heart rate response to hypoxia and HRV parameters (positive correlations with RMSSD, pNN50) and BRS parameters (positive correlation with BRS-CB and negative correlation with BRS-αLF) were found. It suggests that depressed parasympathetic drive (relations with RMSSD and pNN50), and impaired cardiac baroreflex (relation with BRS-CB), especially overactive sympathetic arm of this response (relation with BRS-αLF) are possibly linked to blunted heart rate response from peripheral chemoreceptors. Both depressed autonomic control of heart function and impaired cardiac baroreflex result in reduced cardiac “plasticity” regarding limited ability of the heart to adjust its function to meet the body’s needs and to react on external stressors properly (e.g. hypoxia).

The results of this study seem to support the age-related association between autonomic derangement and deterioration of heart rate response from peripheral chemoreceptors. However, alternative explanations of blunted cardiac response may be considered. Reduced heart rate acceleration in response to hypoxia in the older group may be secondary to exaggerated blood pressure increase with reflex stimulation and response from arterial baroreceptors. However, as baroreflex sensitivity is already deranged in these subjects, this seems unlikely.

Secondly, the possible impact of age-related changes in activity of pulmonary stretch receptor reflex should be considered. It was shown recently that pulmonary stretch receptor feedback contributes to inhibition of baroreflex-induced bradycardia in anaesthetized rats [41]. Remarkable structural and functional alternations in the respiratory system occurring with age [42] could possibly be related to the deterioration of pulmonary stretch receptors and in turn diminished activity of this reflex. As a result, in middle-aged and elderly men, this diminished pulmonary stretch receptor reflex may be unable to inhibit vagal outflow to the heart and therefore fails to increase the heart rate.

Finally, various changes observed in the aging heart (such as lower sensitivity of the β-adrenergic receptors combined with a decreased population of sinoatrial node’s pacemaker cells) may result in limited ability of the heart to respond to external stimuli [43] despite tonic sympathoexcitation [20].

Contrary to the results for heart rate response, there were no relationships between systolic blood pressure response to hypoxia and HRV and BRS parameters in the group of men ≥50 years old and only a few in the men <50 years old. It may indicate that age-related changes in blood pressure response to hypoxia are not related to autonomic derangements. Such a result may be surprising as there is an evidence for an anatomical basis for an interaction between peripheral chemoreceptors and arterial baroreceptors (their afferents converge partially onto the same neurons within the nucleus tractus solitarii [44–46]). In clinical studies, antagonistic baroreflex–chemoreflex interaction (i.e. activation of arterial baroreflex inhibits reflex hemodynamic responses from peripheral chemoreflex and vice versa) was well documented [47, 48]. Lack of statistically significant inverse correlations between individual values of BRS indices and PChS-SBP in our study should be interpreted in this physiological context. In fact, augmented PChS-SBP response to hypoxia in the older group may well be a result of impaired BRS.

Interestingly, recent works showed that there is no simple relation between sympathetic outflow to systemic blood vessels (measured by a microneurography technique) and blood pressure at rest [49]. Probably, other factors such as level of cardiac output, adrenergic sensitivity and rate of nitric oxide synthesis [50] may contribute greatly to tonic blood pressure regulation. It may be hypothesized that these factors are also engaged in phasic responses of blood pressure to various stimuli, such as acute hypoxia.

Heart rate and systolic blood pressure response to hypoxia did not correlate. It stays in line with different pattern of relations with “markers” of autonomic status and regulation found for these responses. A recent study from our laboratory has shown that bilateral carotid body removal in men with systolic heart failure reduced respiratory and systolic blood pressure response to hypoxia, but did not affect cardiac response [22]. Thus, it may be hypothesized that neural substrates for blood pressure and cardiac response to hypoxia are divergent, and the impact of aging on these pathways may also be variable.

As an alternative to the hypothesis of “autonomic derangement”, age-related alterations in blood vessels may promote exaggerated blood pressure increases in response to hypoxia in middle-aged and elderly men. These age-related changes include: stiffening of the blood vessel walls, excessive release of vasoconstrictors with concomitant decline in vasodilator production, and increase in density of α_2_ receptors with sympathetic over-activation [51].

### Study limitations

Some limitations of this study should be emphasized. Clearly, longitudinal observation with intra-individual comparisons would be an ideal design to explore consequences of ageing. To the best of our knowledge, the study of Lhuissier et al. [17] is the only one which was based on a longitudinal, intra-individual comparison study design. The age of 50 years was arbitrarily chosen as a cutoff to divide the study population into two age groups. It was based on previous reports on linear decline in HRV and cardiac BRS with a deflection point being around mid-fifties [11]. As only male subjects were enrolled in the study, we need to acknowledge that our results cannot necessarily be extrapolated to women. All hemodynamic parameters assessed in this study were derived from the Nexfin device, which non-invasively estimates beat-by-beat stroke volume by the instantaneous analysis of the blood pressure waveform [52]. It can be considered a study limitation, as for SV/CO assessment, because invasive methods may be viewed as the “gold standard”. However, the clinically acceptable agreement between the values of hemodynamic parameters obtained using Nexfin device and obtained with direct, invasive methods was confirmed by several studies [52–55]. Additionally, our results should be interpreted cautiously given the large number of statistical tests performed, which increases the chance of false-positive results. It is suggested that chemosensitivity can be divided into: phasic chemosensitivity (referring to reactivity to stimuli) and tonic chemosensitivity (referring to tonic activity of chemoreflex pathways), which are not tightly related [23]. In our study we only evaluated phasic chemosensitivity, thus the possible impact of aging on tonic chemosensitivity (which can be measured by the hyperoxic method [3] or the dopamine infusion method [23]) provide a potentially attractive area to explore.

## Conclusions

The results suggest that the hypothesis of the “autonomic derangement” basis of age-related changes in chemosensitivity may be true for heart rate response from peripheral chemoreceptors, but not for systolic blood pressure response to hypoxia. Moreover, respiratory response from both central and peripheral chemoreceptors seem to be rather independent from sympathovagal balance within the cardiovascular system and barosensitivity.
